# Marriage Laws and Wisconsin’s Experience

**Published:** 1914-03

**Authors:** 


### Marriage Laws and Wisconsin's Experience.

If Wisconsin's Legislature was stupid in passing an impossible statute it should not be condemned for its purposes were commendable. Protection of the home against venereal catastrophy is certainly a worthy cause. Now that the absurdity of the act has been exposed no other than good can come from it. From an educational standpoint it has advanced the cause of eugenics more than any other incident occurring in the year.

The problem simply resolves itself into the question, whether or not the State has the right to safeguard the public health by requiring medical examination for marriage. The public health is the chief concern of the State, and, inasmuch as the venereal plague causes more morbidity and incapacity than all other infectious diseases combined, it would seem that the State would be very derelict in its duty not to extend this protection. This point being settled, it then becomes a question how this could best be done. If all men were imbued with the same high moral principle of right and wrong, simply educating the public would meet the requirements. But, since a large part of every population is without this moral principle it becomes an imperative necessity to enact such laws as will give the State the power to compel this protection.

Such laws would not be an obtrusion on the rights of properly educated citizenship and could but help that class who must be restrained.

The form that such a law should take is yet not so self-evident. The Wisconsin incident has emphasized the ridiculousness of trying to force a statute that is impossible of compliance. It is safe to say that with our present knowledge of eugenics it would be very hasty to include in such laws questions of heredity, at least so far as compelling certain measures to prevent procreation. But as a hygienic safeguard there could be no valid objection to making the law include the acute venereal diseases, imbecility and insanity. We would go still further and advocate a law that would make it a penal offense for one person to infect another with any venereal disease. The State can best accomplish this protection by providing suitable means for the scientific treatment of venereal diseases and the dissemination of prophylactic measures. In the main, it rests with society to practice a positive eugenics rather than urge the negative side. Foster an education that will teach *self-preservation,* an education that will urge parents to safeguard their children's health and happiness. Out of such instruction as this proper laws will be evolved.

